# Case report: Biphasic autonomic response in decompression sickness: HRV and sinoatrial findings

**DOI:** 10.3389/fphys.2025.1605779

**Published:** 2025-04-29

**Authors:** Gerald Schmitz

**Affiliations:** Centro de Medicina Hiperbarica OHB, Hyperbaric Medical Service, San Jose, Costa Rica

**Keywords:** decompression sickness, sinoatrial dysfunction, bradycardia, autonomic dysfunction, heart rate variability, hyperbaric oxygen therapy, principal dynamic mode, Poincaré plot

## Abstract

**Background:**

Decompression sickness (DCS) may involve neurological and cardiovascular systems, but cardiac autonomic dysfunction is rarely documented. Heart rate variability (HRV) can provide insight into autonomic modulation in such cases, particularly when incorporating advanced nonlinear and dynamic techniques.

**Case:**

We present a 35-year-old recreational diver who developed neurological DCS and persistent bradycardia following multiple consecutive dives. Neurological symptoms resolved with hyperbaric oxygen therapy (HBOT), but bradyarrhythmias persisted, prompting continuous monitoring.

**Methods:**

HRV was assessed using time-domain, frequency-domain, nonlinear, and dynamic analyses during HBOT and over two 24-h Holter recordings. Principal Dynamic Mode (PDM) analysis was employed to characterize autonomic control dynamics beyond conventional spectral markers.

**Results:**

During HBOT, the patient exhibited pronounced parasympathetic activity (RMSSD: 243 m; HF power: 8,656 m^2^; SD1: 172 m). Post-treatment, a shift toward sympathovagal imbalance was observed, with the LF/HF ratio rising from 1.53 to 3.80. Despite high total HRV power (38,549 m^2^ during HBOT), SD1/SD2 ratio declined from 0.52 to 0.12, suggesting selective vagal withdrawal. PDM analysis showed a low PDM2/PDM1 ratio (0.42), consistent with preserved beat-to-beat vagal responsiveness but impaired long-range autonomic integration.

**Conclusion:**

This case illustrates a biphasic autonomic pattern in DCS—initial parasympathetic dominance followed by sympathetic tilt and desynchronization. Advanced nonlinear and dynamic HRV analysis revealed regulatory disturbances not captured by traditional methods, supporting its role in post-dive assessment and autonomic monitoring.

## Introduction

Decompression sickness (DCS), a clinical syndrome arising from reduced ambient pressure, is a known risk in diving, typically manifesting within 24 h of ascent from depths as shallow as 20 feet of seawater. Known as “the bends,” DCS results from nitrogen bubble formation in the bloodstream and tissues due to rapid pressure decreases. These bubbles can obstruct blood flow, trigger inflammation, and cause mechanical tissue damage, leading to symptoms that vary by location and severity (Mitchell, 2024). Manifestations range from joint pain, skin rash, and fatigue to severe neurological symptoms such as motor weakness, ataxia, and life-threatening conditions like pulmonary edema and shock. Prompt recognition and treatment are critical to minimize long-term complications, highlighting the need for increased awareness and safe diving practices (Moon, 2014).

Although cardiac manifestations of DCS have been described (Mendez et al., 2017; Brauzzi et al., 2013; Shitara et al., 2019; Hughes et al., 2023), arrhythmias and autonomic disorders are rarely reported (Halpern and Greenstein, 1981). This case describes a rare presentation of neurological DCS associated with sinoatrial node dysfunction and bradycardia.

Heart rate variability (HRV) is a non-invasive marker of autonomic modulation and has been extensively applied in the study of cardiovascular and neurological disorders. Time-domain metrics such as SDNN reflect overall variability, while RMSSD and pNN50 are markers of parasympathetic (vagal) activity. Frequency-domain analysis decomposes variability into high-frequency (HF, 0.15–0.4 Hz), associated with parasympathetic tone, and low-frequency (LF, 0.04–0.15 Hz), linked to baroreflex activity and both autonomic branches. The LF/HF ratio is widely used as an index of sympathovagal balance, although not a pure measure of sympathetic activity (Heart Rate Variability, 1996; Billman, 2013). Nonlinear measures such as the Poincaré plot (SD1/SD2 ratio) offer additional insight into autonomic complexity and balance.

However, traditional HRV analysis may fail to detect subtler regulatory disturbances, especially when autonomic inputs are nonlinear or nonstationary, as in acute stress or injury. To address this, dynamic modeling techniques such as Principal Dynamic Mode (PDM) analysis have been proposed. PDM decomposes the dynamic relationship between autonomic input and heart rate output, identifying distinct sympathetic and parasympathetic control modes (Zhong et al., 2004). This approach has been used in experimental models of DCS (Bai et al., 2013), but has not yet been applied in human clinical cases.

We report the first known case of biphasic autonomic disturbance in a diver with neurological DCS, evaluated through multimodal HRV including PDM. The case highlights the complexity of autonomic recovery after DCS and demonstrates the added value of advanced analytic tools in diving medicine.

## Case report

Written informed consent was obtained from the patient for the publication of anonymized data and figures. The patient provided written informed consent for the publication of this case report, including anonymized clinical data and heart rate variability analysis results. This case report adheres to the ethical principles of the Declaration of Helsinki and local law on biomedical research (Law # 9234).

A 35-year-old male recreational diver with 71 lifetime dives, rescue certification, and chronic cannabinoid use presented with neurological and cardiovascular symptoms following six to seven consecutive days of diving (two to four dives/day, 14–20 m of seawater [msw], 40–51 min, on air). He had no known cardiovascular disease, no family history of arrhythmia, and no use of prescribed medications. He reported regular cannabis use over the preceding year, approximately 3–5 times weekly, primarily in the evenings. He was otherwise physically active, non-smoker, and denied alcohol or stimulant use.

The final dive reached 187 kPa (equivalent to ∼18 m) for 14 min, with a controlled ascent rate <9 msw/min. Approximately 10 min post-surfacing, he experienced transient lightheadedness and unsteadiness. Five hours later, he developed vertigo, palpitations, and left leg paresthesia. At initial evaluation, he was hemodynamically stable, alert, and oriented, with a heart rate (HR) of 83 beats per minute (bpm), blood pressure of 122/74 mmHg, and SpO_2_ of 98% on room air.

Neurological examination revealed decreased sensation over the anterior left thigh and mild weakness of the left psoas (4/5). Reflexes were symmetric, Babinski was negative bilaterally, and coordination was intact. Cardiovascular and respiratory examinations were unremarkable. An electrocardiogram (ECG) performed 12 h post-dive showed sinus bradycardia (53 bpm) with intermittent RR interval prolongation without P-wave abnormalities, suggesting second-degree sinoatrial node exit block. No ectopic beats or structural abnormalities were present. Similar conduction abnormalities have been described in severe cases of decompression sickness (Leitch and Hallenbeck, 1985).

He was transferred to the hyperbaric unit 16 h after surfacing. On arrival, his HR was 43 bpm. Hyperbaric oxygen therapy (HBOT) was initiated using a USN-TT6 protocol in a Sechrist H3300 chamber. The session included: compression to 284 kPa (18,3 msw), oxygen breathing for 20 min at 284 kPa, a series of alternating air breaks and oxygen periods, followed by gradual decompression to 193 kPa, with oxygen periods of 60 min alternated with air break periods. The total chamber time was approximately 285 min.

Neurological symptoms resolved within 20 min at 284 kPa. However, continuous ECG monitoring during HBOT showed sustained bradycardia (HR 38–43 bpm). No medication or sedatives were administered.

A 24-h Holter monitor (Holter 1) was initiated 22 h post-HBOT due to persistent fatigue and perceived instability. The recording revealed sinus bradycardia (average HR 46 bpm), intermittent sinoatrial block, and Mobitz I (Wenckebach) atrioventricular block. Echocardiography revealed structurally normal cardiac anatomy with preserved ejection fraction (EF: 62%).

A second Holter on Day 4 (Holter 2) showed normalization of HR (average 67 bpm), no further conduction disturbances, and complete resolution of symptoms.

### Patient perspective

The patient later reported: “*I was surprised that my heart rate stayed so low after I felt better. I’m glad they monitored me closely, because I did not feel right even after the chamber.*”

## Methodology

HRV analysis was conducted using custom Python scripts following international recommendations (Heart Rate Variability, 1996). R-R interval data were obtained from two sources:

1. During HBOT: Continuous ECG monitoring via the Mindray BeneVision N19, exported at 500 Hz and processed offline to extract R-peaks. A 60-min artifact-free segment at the beginning of the treatment phase at 284 kPa was used.
2. Post-HBOT: 24-h Holter ECG recordings (Holter 1: Day 2; Holter 2: Day 4) acquired using GE SEER Light, 3-channel devices, reviewed for artifacts by two independent clinicians.

### Artifact handling and preprocessing

Signals were visually inspected and filtered using IQR-based outlier rejection (based on local RR differences). Non-sinus beats and artefacts were excluded from analysis. No interpolation was applied unless consecutive RR values were missing.

### Software

All analyses were performed in Python 3.9 using NumPy, SciPy, and NeuroKit2. Spectral analysis used Welch’s method (Hanning window, 256-sample length, 50% overlap). R-R data were resampled at 4 Hz for frequency-domain and nonlinear processing.

**Time-domain metrics** included:

• **SDNN** (ms): Standard deviation of normal R-R intervals (global variability),
• **RMSSD** (ms): Root mean square of successive differences (short-term vagal activity),
• **pNN50** (%): Proportion of RR differences >50 m (parasympathetic marker).

Frequency-domain metrics:

• **Total Power (TP, ms** ^**2**^ **)**: Overall spectral power <0.4 Hz,
• **VLF (<0.04 Hz)**: Long-term, undefined influences,
• **LF (0.04–0.15 Hz)**: Baroreflex/sympathetic-parasympathetic composite,
• **HF (0.15–0.4 Hz)**: Parasympathetic modulation (respiratory-linked),
• **LF/HF ratio**: Index of **sympathovagal balance**, not pure sympathetic tone (Billman, 2013).

**Nonlinear analysis** was performed using Poincaré plots:

• **SD1 (ms)**: Instantaneous beat-to-beat variability (vagal),
• **SD2 (ms)**: Long-term HRV,
• **SD1/SD2 ratio**: Autonomic coordination and desynchronization index.

Note: Although normalized LF and HF units were computed, they were excluded from reporting due to interpretive redundancy and reviewer alignment.

PDM analysis was used to model the dynamic response of heart rate to autonomic inputs. Following the method by Zhong et al. (Zhong et al., 2004), RR signals were modeled using Laguerre expansion of kernels to capture impulse response dynamics. Key steps included:

• Generation of Laguerre basis functions (α = 0.8, L = 6),
• Estimation of input-output kernels relating RR changes to autonomic drives,
• Singular Value Decomposition (SVD) of kernel matrices to extract dominant modes,
• Identification of PDM1 (fast, vagal component) and PDM2 (slower, sympathetic/long-range modulation),
• Computation of PDM2/PDM1 ratio as an index of autonomic integration balance.

This method enables detection of delayed or impaired autonomic control not evident in linear metrics and has been validated in DCS animal models (Bai et al., 2013).

Table 1 summarizes the metrics and their physiological interpretation.

**TABLE 1:** Summary of heart rate variability (HRV) metrics and their typical physiological interpretations.

Domain	Metric	Physiological interpretation
Time-domain	SDNN (ms)	Standard deviation of normal R-R intervals; reflects overall autonomic variability
	RMSSD (ms)	Root mean square of successive differences; reliable marker of parasympathetic (vagal) activity
	pNN50 (%)	Percentage of R-R intervals differing by >50 m; indicative of short-term vagal modulation
Frequency-domain	Total Power (ms^2^)	Overall HRV power across <0.4 Hz; includes all autonomic influences
	VLF (<0.04 Hz)	Long-term HRV fluctuations; may relate to thermoregulation and hormonal rhythms
	LF (0.04–0.15 Hz)	Mixed sympathetic and parasympathetic influences; sensitive to baroreflex modulation
	HF (0.15–0.4 Hz)	Parasympathetic (vagal) activity, especially respiratory-linked
	LF/HF ratio	Index of sympathovagal balance (not a direct measure of sympathetic tone) [8-1]
Nonlinear	SD1 (ms)	Beat-to-beat variability; correlates with RMSSD and vagal tone
	SD2 (ms)	Long-term variability; reflects combined autonomic influences
	SD1/SD2 ratio	Coordination between short- and long-term variability; low values may reflect autonomic imbalance
Dynamic (PDM)	PDM1	Principal dynamic mode representing fast (vagal) control response
	PDM2	Slower mode reflecting delayed (often sympathetic) modulation
	PDM2/PDM1 ratio	Indicator of dynamic integration and regulatory balance; low values may suggest desynchronization

This table outlines the principal HRV metrics used in this study, classified by analysis domain. Interpretations reflect prevailing consensus in the HRV literature, with time-domain and high-frequency (HF) metrics reliably associated with parasympathetic modulation. Some frequency-domain parameters (e.g., low-frequency [LF], LF/HF ratio) and very-low-frequency (VLF) components are known to reflect mixed autonomic inputs and baroreflex activity rather than pure sympathetic output. Principal Dynamic Mode (PDM) parameters are derived from system identification models and represent fast (PDM1) and delayed (PDM2) autonomic dynamics. Interpretations should be contextualized within each recording’s duration, conditions, and analytic assumptions.

## Results

HRV analysis revealed a biphasic autonomic response across the three monitoring periods: during HBOT (oxygen phase at 284 kPa), Holter 1 (22 h post-HBOT), and Holter 2 (Day 4 post-treatment). Table 2 summarizes the time-domain, frequency-domain, and nonlinear HRV metrics.

**TABLE 2:** Heart rate variability metrics across three time points: During hyperbaric oxygen therapy (HBOT), Holter 1 (Day 2), and Holter 2 (Day 4).

HRV metric	During HBOT	Holter 1 (Day 2)	Holter 2 (Day 4)
Average HR (bpm)	42	46	67
SDNN (ms)	263	235	235
RMSSD (ms)	243	95	55
pNN50	66	46	24
Total power (ms^2^)	38549	15866	10020
LF power (ms^2^)	13,277	2,104	2,027
HF power (ms^2^)	8,656	554	620
LF/HF ratio	1.53	3.80	3.26
SD1 (ms)	172	67	39
SD2 (ms)	329	341	330.0
SD1/SD2 ratio	0.52	0.20	0.12
PDM1	24.5		
PDM2	10.3		
PDM2/PDM1	0.42		

This table summarizes time-domain, frequency-domain, and nonlinear HRV, parameters recorded during HBO, therapy and two subsequent 24-h Holter monitors. The data reflect a biphasic autonomic response, including initial parasympathetic dominance followed by vagal withdrawal.

During HBOT, the patient exhibited marked parasympathetic activity, with RMSSD of 243 m, pNN50 of 66%, and SD1 of 172 m. High-frequency (HF) power was elevated at 8,656 m^2^. These findings were derived from a 60-min continuous R-R interval segment acquired during exposure at 284 kPa.

Following treatment, Holter 1 (24-h recording on Day 2) revealed a decline in parasympathetic indices: RMSSD decreased to 95 m, pNN50 to 46%, and HF power to 554 m^2^. LF power increased to 2,104 m^2^, resulting in a rise in LF/HF ratio from 1.53 (HBOT) to 3.80, indicating a shift in sympathovagal balance.

By Holter 2 (Day 4), further parasympathetic decline was observed (RMSSD: 55 m, pNN50: 24%, HF: 620 m^2^), while LF power remained stable (2,027 m^2^), and LF/HF ratio decreased slightly to 3.26.

**FIGURE 1:**
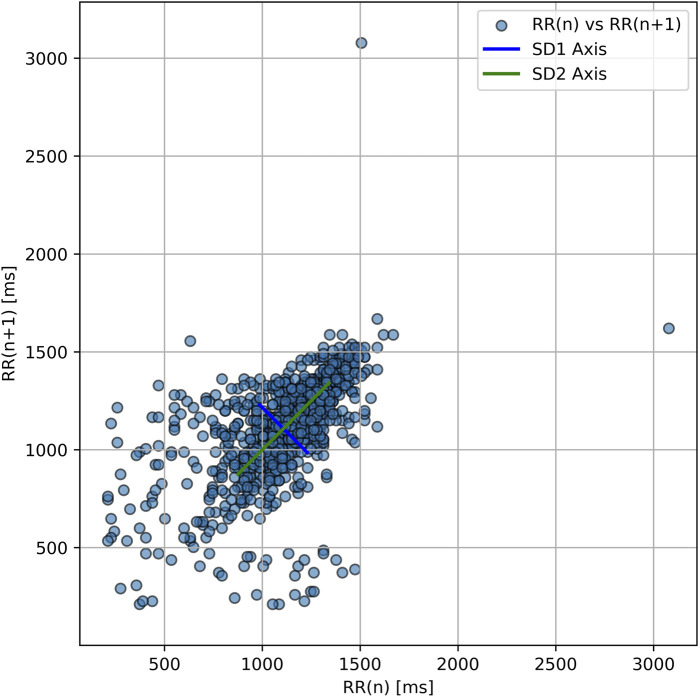
Poincaré plot of RR intervals during hyperbaric oxygen therapy. Each point represents one RR_n_ vs. RR_n+1_ interval. The ellipse orientation shows strong overall variability with elongation along the SD2 axis and a narrower SD1 axis. This geometry reflects a low SD1/SD2 ratio (∼0.52), indicating a regulatory imbalance in autonomic function—consistent with persistent autonomic desynchronization.

The SD1/SD2 ratio, a nonlinear index of autonomic coordination, progressively declined across sessions (0.52 → 0.20 → 0.12), suggesting increasing autonomic desynchronization despite relatively stable long-term variability (SD2 ∼330 m throughout) (Figure 1).

Principal Dynamic Mode (PDM) analysis revealed a preserved fast vagal response (PDM1 = 24.5), but a low slow-mode component (PDM2 = 10.3), yielding a PDM2/PDM1 ratio of 0.42. This pattern is consistent with impaired long-range or delayed autonomic regulation.

Table 3 compares this patient’s HRV values to published findings in healthy divers, untreated DCS cases, and known acute and chronic cannabis exposure profiles.

**TABLE 3:** Comparison of the Diver’s HRV metrics with healthy post-dive profiles, untreated decompression sickness (DCS), and cannabis use.

HRV metric	Healthy diver after dive	Untreated DCS	This case (diver)	Acute cannabis use	Chronic cannabis use
HR (bpm)	↓ HR or normal	Variable	↓ (Bradycardia: 38–46 bpm)	↑	↔ or ↑
Total Power (TP) (ms^2^)		↓	↑ (38,549 → ↓ 10,020)	↓	↔ or ↑
RMSSD (ms)		↓	243 → 55	↓	↑
HF power (ms^2^)	↓	↓	Elevated during HBOT, declined post	↓	↑
LF/HF ratio	↑ (∼0.8–1.03)	↑	1.53 → 3.80 → 3.26	↑	↓
SD1/SD2 ratio	Not reported	↓	∼0.52 (low)	↓	↑

This table compares the diver’s HRV profile to known patterns in healthy divers, DCS, and acute and chronic cannabinoid exposure, based on referenced literature. Only metrics supported by published evidence are included.

## Discussion

This case highlights a rare presentation of decompression sickness (DCS) involving transient sinoatrial and atrioventricular conduction disturbances, accompanied by a biphasic autonomic pattern. Using multimodal heart rate variability (HRV) analysis, we identified a distinct evolution from parasympathetic dominance during hyperbaric oxygen therapy (HBOT) to progressive vagal withdrawal and sympathovagal imbalance during post-treatment recovery.

Traditional time- and frequency-domain metrics suggested robust vagal tone during HBOT, consistent with the known vagotonic effects of hyperbaric oxygen in healthy individuals (Lafère et al., 2021; Lund et al., 2003; Lund et al., 1999). As recovery progressed, vagal indices such as RMSSD, SD1, and HF power declined, while the LF/HF ratio increased. This shift reflects a rebalancing of autonomic tone, although LF/HF should be interpreted as an index of sympathovagal modulation, not pure sympathetic activity (Billman, 2013).

Nonlinear HRV parameters further illustrated regulatory imbalance. The SD1/SD2 ratio declined steadily after HBOT, suggesting increasing autonomic desynchronization. Importantly, SD2 remained stable, indicating that long-term variability was preserved even as vagal short-term control deteriorated.

Principal Dynamic Mode (PDM) analysis provided unique insights beyond conventional HRV. While beat-to-beat vagal responsiveness (PDM1) was preserved, the attenuated delayed response (PDM2) and low PDM2/PDM1 ratio indicated impaired long-range modulation, potentially reflecting disrupted baroreflex buffering or central autonomic integration.

This autonomic desynchronization pattern is consistent with stress-related HRV changes reported during decompression profiles in experimental and diver studies (Schirato et al., 2020). These findings also parallel animal studies of DCS-induced autonomic dysregulation (Bai et al., 2013; Bai et al., 2009).

Although speculative, the observed pattern may result from bubble-induced endothelial dysfunction or circulating microparticles impairing neurovascular integrity (Yang et al., 2013; Thom et al., 2015). The persistence of arrhythmic patterns and regulatory collapse post-HBOT warrants attention, especially in divers presenting with delayed or non-resolving symptoms.

The role of cannabis use in this case is likely minor. While chronic use may elevate RMSSD or baseline vagal tone (Pabon et al., 2022; Schmid et al., 2010), acute exposure typically induces tachycardia and sympathetic activation (Berry et al., 2017). Given the bradycardia and conduction blocks observed here, DCS-related autonomic disruption remains the more plausible cause. Nonetheless, the cannabis literature on HRV is limited and inconclusive.

Clinically, this case suggests that advanced HRV analysis, including nonlinear and dynamic techniques, could assist in identifying autonomic instability post-dive—even when conventional metrics appear within range. Parameters such as the SD1/SD2 ratio or PDM profiles may serve as early markers of dysautonomia or delayed recovery, guiding prolonged observation or tailored return-to-dive decisions.

While a sympathetic tilt has been described in healthy divers during and after immersion (Berry et al., 2017), animal models of decompression sickness (DCS) suggest a predominance of parasympathetic activity during acute decompression stress (Bai et al., 2009). Various arrhythmias, including sinus and atrioventricular conduction abnormalities, have been reported both in asymptomatic divers (Gunes and Cimsit, 2017; Bosco et al., 2014) and in confirmed DCS cases (Kizer, 1980), although they appear relatively rare and are not consistently documented in the literature. In this context, heart rate variability (HRV) analysis may offer a novel and non-invasive window into autonomic alterations associated with DCS. Although HRV measurement standards exist (Shaffer and Ginsberg, 2017), DCS-specific patterns, cutoff values, and recovery trajectories remain to be clearly defined (Schaller et al., 2021).

## Limitations

This report is limited by the absence of respiratory rate monitoring, which limits interpretation of HF variability. The HBOT HRV segment (∼60 min) is shorter than the 24-h Holter data, which may affect metric comparability. Pre-dive HRV baseline was not available, preventing individualized deviation analysis. While artifact handling was conservative, no formal interpolation or ECG-based beat classification algorithm was used. PDM findings, while physiologically meaningful, should be interpreted in the context of model assumptions and limited clinical validation in DCS.

## Conclusion

This case illustrates the potential for persistent autonomic dysfunction following neurological decompression sickness (DCS), even in the absence of structural cardiac abnormalities. The observed biphasic autonomic pattern—parasympathetic dominance during hyperbaric oxygen therapy (HBOT), followed by post-treatment vagal withdrawal and progressive desynchronization—was evident through multiple HRV domains.

Conventional time- and frequency-domain metrics captured broad changes in autonomic tone, while nonlinear measures and Principal Dynamic Mode (PDM) analysis revealed additional subtleties in autonomic regulation. Notably, the decline in SD1/SD2 ratio and low PDM2/PDM1 ratio pointed to impaired autonomic integration and long-range modulation, consistent with delayed recovery or baroreflex dysfunction.

These findings suggest that advanced HRV analysis, including nonlinear and dynamic techniques, may offer added value in post-dive evaluation—particularly in symptomatic divers or those with suspected dysautonomia. Such tools could support individualized monitoring strategies, guide follow-up intensity, or inform return-to-dive decisions.

While this report cannot establish causality, it reinforces the need for comprehensive autonomic assessment in diving medicine. Future studies should explore the prognostic significance of HRV dynamics and the role of modeling-based metrics in clinical decision-making.

## Data Availability

The datasets presented in this article are not readily available because raw data are contained within the clinical database. Requests to access the datasets should be directed to gschmitzg@gmail.com.

## Abbreviations

DCS, Decompression Sickness; HBOT, Hyperbaric Oxygen Therapy; HR, Heart rate; HRV, Heart rate variability; TP, Total power; SDNN, Standard deviation of normal R-R intervals; RMSSD, Root mean square of successive differences; PDM, Principal Dynamic Mode; VLF, Very Low Frequency; LF, Low Frequency; HF, High Frequency; PDM1, First principal dynamic mode; PDM2, Second principal dynamic mode; msw, Meters of Seawater.
